# One-step purification and immobilization of extracellularly expressed sortase A by magnetic particles to develop a robust and recyclable biocatalyst

**DOI:** 10.1038/s41598-017-06856-y

**Published:** 2017-07-26

**Authors:** Xinrui Zhao, Haofei Hong, Xiaozhong Cheng, Shaozhong Liu, Tao Deng, Zhongwu Guo, Zhimeng Wu

**Affiliations:** 1 Key Laboratory of Carbohydrate Chemistry & Biotechnology, Ministry of Education, School of Biotechnology, Jiangnan University, Wuxi, China; 2 Department of Chemistry, University of Florida, 214 Leigh Hall, Gainesville, Florida 32611 United States of America

## Abstract

Sortase A (SrtA) is a transpeptidase widely used to site-specifically modify peptides and proteins and shows promise for industrial applications. In this study, a novel strategy was developed for constructing immobilized-SrtA as a robust and recyclable enzyme via direct immobilization of extracellularly expressed SrtA in the fermentation supernatant using magnetic particles. Efficient extracellular SrtA expression was achieved in *Escherichia coli* through molecular engineering, including manipulation of the protein transport pathway, codon optimization, and co-expression of molecular chaperones to promote expressed SrtA secretion into the medium at high levels. Subsequently, a simple one-step protocol was established for the purification and immobilization of SrtA containing a His-tag from the fermentation supernatant onto a nickel-modified magnetic particle. The immobilized SrtA was proved to retain full enzymatic activity for peptide-to-peptide ligation and protein modification, and was successfully reused for five cycles without obvious activity loss.

## Introduction

Enzymes are widely used biocatalysts for many applications in chemical, pharmaceutical and material industries and biomolecule modification, among others^1–8^. Although biocatalyst has been considered a green and sustainable approach, some inherent drawbacks of particular enzymes, such as the difficult availability, poor stability, high cost and difficulty in recovery and recycling, have hampered their wider applications. Therefore, the enzyme immobilization technology has recently attracted great attention, as this technique offers a practical approach to overcome the aforementioned disadvantages^9–11^.

In particular, the use of magnetic particles as a solid support for enzyme immobilization has been extensively explored in recent years because these materials provide not only a high surface/volume ratio and high dispersity but also easy and fast magnetic separation^12, 13^. Many approaches, including covalent attachment, noncovalent adsorption, entrapment and cross-linking, have been developed to successfully immobilize enzymes onto magnetic particles^14^. However, in most cases, challenging and costly pre-purification to obtain pure enzymes is required before immobilization^15–17^. Thus, to further simplify constructing immobilized biocatalysts with potential industrial applications, an improved approach based on direct immobilization extracellularly expressed enzymes in the fermentation supernatant was recently developed. For example, a magnetic particle-immobilized lipase was generated from secretory protein in the fermentation supernatant via His-tag affinity and was demonstrated to be an active and recyclable immobilized biocatalyst for biodiesel production from waste grease^18^. Inspired by these results; we envisioned that this approach might be extended to immobilize biocatalysts for challenging biomolecular conjugation reactions.

Sortase A (SrtA) is a membrane bound transpeptidase that anchors surface proteins to bacterial cell wall through a “cell wall sorting process”^19^. After the successful expression of a biologically active, water soluble, truncated SrtA in *Escherichia coli* from *Staphylococcus aureus* ^20^, SrtA-mediated ligation (SML) technology has developed into a powerful tool to site-specifically modify biomolecules of interest^21–24^. For example, SML has been used for peptide/protein and living cell surface labeling^25–28^, protein-to-protein fusion^29–31^, antibody-drug conjugation^32–34^, peptide and protein cyclization^35–38^, GPI-linked peptide and protein synthesis^39–41^, immobilization of biocatalysts to an artificial surface^42, 43^, *in vivo* protein tagging^44^, and thioester preparation^45^, among others^30, 46^. Recently, SrtA variants with improved kinetics have been generated with yeast display technology^47^ and a complementary approach including error-prone PCR and saturated mutagenesis^32^.

However, several bottlenecks have limited SML in industry implementations. First, SrtA was expressed in the cytoplasm, which requires nontrivial cell disruption and costly purification to obtain pure and active enzyme^20^. Second, the SrtA expression yield is rather low, yielding up to 77 mg/L at most^48^. In addition, SrtA-mediated ligation usually needs a near equivalent of enzyme, leading to challenging downstream purification of the ligation product from the reaction mixture with SrtA^41, 49^. To overcome these issues, recyclable immobilized SrtA for SML was recently developed. For example, the Beck-Sickinger group immobilized SrtA onto an acrylamide-PEG co-polymer (PEGA) resin through Cu(I) catalyzed “click chemistry” to generate an immobilized biocatalyst with low enzymatic activity^50^. Pentelue and coworkers developed flow-based SML, in which SrtA was immobilized onto Ni-NTA agarose resin using a His-tag^16^. This protocol was particularly useful for preparing protein conjugates inaccessible by traditional solution batch ligation when low nucleophile concentrations were used. Ploegh and coworkers investigated the covalent immobilization of SrtA onto cyanogen bromide-activated Sepharose beads using a chemical approach^49^. More recently, Francis and coworkers developed an approach to capture and recycle lithocholic acid-modified SrtA by means of β-cyclodextrin-functionalized Sepharose beads, which can be recycled for multiple rounds. However, preparing lithocholic acid-modified SrtA is complicated and requires three more steps, including protection of the catalytic site cysteine*, in vitro* oxidative coupling and deprotection^17^. Nevertheless, SrtA or modified SrtA was expressed in an intracellular manner in *E. coli*, which require challenge and expensive downstream bioprocess to obtain pure and active SrtA from the cell lysate for immobilization.

Herein, we present a novel strategy to construct robust and recyclable immobilized SrtA by combining extracellular SrtA expression and its direct immobilization from the fermentation supernatant by magnetic particles and an affinity tag. Specifically, technologies including protein transport pathway manipulation, codon optimization and molecular chaperone co-expression were adopted to promote the expression of recombinant SrtA with a His-tag in *E. coli* and its secretion into medium at high levels. One-step immobilization and purification of the expressed SrtA from the culture medium was performed using iminodiacetic acid (IDA)-nickel modified magnetic particles. The magnetic particle immobilized SrtA (MPI-SrtA) was demonstrated to retain full enzymatic activity and be recyclable without obvious activity loss.

## Results and Discussion

### Extracellular expression of SrtA in *E. coli*

To realize direct immobilization of SrtA, high-level secretory expression of SrtA was one of the key issues, whereas SrtA had only been expressed in the cytoplasm of *E. coli* ^20^. To direct SrtA transfer from the cytoplasm to the periplasm and then into the culture medium, we genetically engineered SrtA to carry a signal peptide in the host *E. coli* strain, such that after the SrtA precursor was expressed in the cytoplasm, it was recognized and translocated by transport proteins to the periplasm, where signal peptides were cleaved by the corresponding peptidases, allowing SrtA secretion into the culture medium through nonspecific periplasmic leakage.

**Figure 1:**
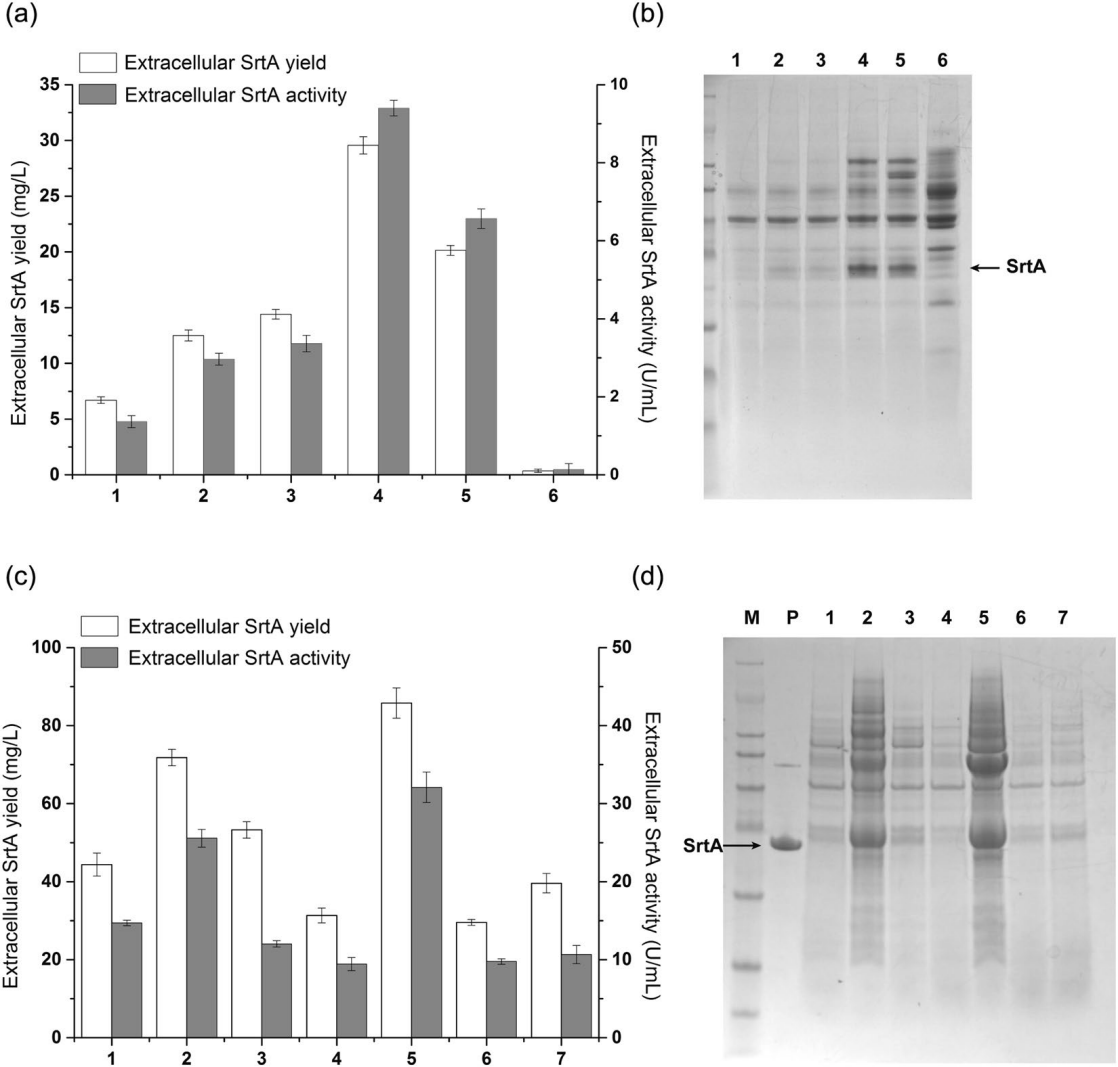
(**a**) Expression level and enzyme activity of secreted SrtA mediated by signal peptides in *E. coli*; 1: FdnG, 2: OmpA, 3: TorA, 4: PelB, 5: DmsA, and 6: control (without signal peptide). (**b**) SDS-PAGE of culture supernatant from *E. coli* BL21 (DE3) encoding signal peptides; lane M: protein marker, lanes 1–6: FdnG, OmpA, TorA, PelB, DmsA, and control (without signal peptide). (**c**) Expression level and enzymatic activity of secreted SrtA including the PelB signal peptide with codon optimization and molecular chaperone co-expression in *E. coli*; 1: GroES/GroEL, 2: Tig, 3: GroES/GroEL/DnaK/DnaJ/GrpE, 4: DnaK/DnaJ/GrpE, 5: GroES/GroEL/Tig, 6: control (without codon optimization and molecular chaperones), and 7: codon optimization only. (**d**) SDS-PAGE of culture supernatant from *E. coli* BL21 (DE3) encoding signal peptides with or without codon optimization and molecular chaperone co-expression; lane M: protein marker, lane P: purified SrtA, lane 1–7: GroES/GroEL, Tig, GroES/GroEL/DnaK/DnaJ/GrpE, DnaK/DnaJ/GrpE, GroES/GroEL/Tig, control (without codon optimization and molecular chaperones), and codon optimization only. All of the experiments were performed in triplicate, and the data are the mean values with the standard deviation.

Two major secretory pathways, Sec-dependent secretion (Sec) and twin-arginine translocation (Tat), were selected to mediate the extracellular expression of SrtA, because of their clear secretion mechanisms and wide applications in extracellular protein expression in *E. coli* ^51, 52^. Therefore, five signal peptides, PelB and OmpA of the Sec pathway and TorA, DmsA, and FdnG of the Tat pathway, were screened using a trial-and-error approach. After the five recombinant plasmids pET22b-FdnG-Δ59-SrtA, pET22b-OmpA-Δ59-SrtA, pET22b-TorA-Δ59-SrtA, pET22b-PelB-Δ59-SrtA, and pET22b-DmsA-Δ59-SrtA were constructed, they were transformed into *E. coli* BL21 (DE3) cells. The recombinant cells were cultured in a shaking flask for 36 h, and the enzymatic activity in the culture medium was measured using a specific FRET substrate (Dabcyl-QALPETGEE-Edans). As shown in Fig. 1a, the extracellular enzymatic activity titers of SrtA from these five recombinant *E. coli* were 1.5, 3.1, 3.6, 9.4 and 6.8 U/mL, respectively. Thereafter, SrtA in the media was purified by Ni-resin affinity chromatography to get 6.7, 12.5, 14.9, 30.2, 20.6 mg/L proteins, respectively, which were consistent with the enzymatic activity results. For control, the culture medium of *E. coli* with SrtA lacking the signal peptides showed only trace amount of enzymatic activity. The SDS-PAGE results of these culture media are shown in Fig. 1b and indicated that both the Sec and Tat pathways were capable of promoting SrtA secretion into the fermentation supernatant with the aid of signal peptides. Among them, the *E. coli* strain engineered with plasmid pET22b harboring the PelB signal peptide exhibited the highest extracellular enzymatic activity and SrtA protein concentration.

Codon optimization is a frequently used strategy to enhance protein production in *E. coli* systems by significantly improving target gene expression^53^. Therefore, we analyzed the SrtA gene sequence by computing codon pair preference, and seven rare codons, including Ile158 (ATA → ATT), Arg99, Arg159 (AGA → CGT), Leu110, Leu169 (CTA → CTG), Gly90, and Gly119 (GGA → GGC), were identified and then site-mutated into the corresponding relatively biased codons for *E. coli* usage (Table SI3). *E. coli* JM109 cells were transformed with the mutated plasmid (pET22b-PelB-Δ59-SrtA*), and the transformants were confirmed by DNA sequencing. The correct plasmid was transformed into *E. coli* BL21 (DE3) cells for SrtA expression. The extracellular protein concentration and SrtA enzymatic activity were increased by 1.40-fold (to 42.3 mg/L) and 1.24-fold (to 11.7 U/mL), respectively (Fig. 1c, column 7).

Co-expression of molecular chaperones with the target gene is another strategy to improve the biological activity and solubility of enzyme in both the cytoplasm and periplasm by preventing protein aggregation^54^. Therefore, to further enhance the extracellular expression and enzymatic activity of SrtA, the *E. coli* BL21 (DE3) cells with pET22b-Δ59-SrtA* were re-transformed with one of the following commercial plasmids, pKJE7, pTf16, pGro7, pG-KJE8, and pG-TF2, encoding the following molecular chaperones, GroES/GroEL, Tig, GroES/GroEL-DnaK/DnaJ/GrpE, DnaK/DnaJ/GrpE, and GroES/GroEL/Tig, respectively. The results are presented in Fig. 1c and d. When SrtA was recombinantly expressed with the plasmid pG-KJE8 co-expressing the DnaK/DnaJ/GrpE chaperones, the extracellular SrtA expression level and enzymatic activity decreased slightly (Fig. 1c, column 4). Co-expression of the plasmids pKJE7 or pGro7 with SrtA increased the extracellular SrtA concentration marginally (Fig. 1c, column 1, 3). Alternatively, co-expression of the pTf16 plasmid encoding the molecular chaperone Tig increased the extracellular SrtA concentration and enzymatic activity by 2.46-fold (to 74.2 mg/L) and 2.82-fold (to 26.5 U/mL), respectively (Fig. 1c, column 2). It is also interesting to observe that co-expression of pG-TF2, which encodes both GroES/GroEL and Tig under the same conditions, demonstrated maximal activity (34.0 U/mL) and expression level (89.8 mg/L) for extracellular SrtA (Fig. 1c, column 5). After purification of His-tagged SrtA with the nickel resin and analysis by SDS-PAGE, a clear protein band was observed at 17.6 KDa, which agrees with the expected MW of Δ59-SrtA. This result indicates that suitable molecular chaperones might enhance the biological activity and solubility of SrtA expressed in the cytoplasm and increases SrtA secretion into the periplasm and culture medium. It is noteworthy that this is the first report examining the extracellular expression of SrtA in *E. coli*, and the current expression yield was higher than the highest SrtA yield achieved with intracellular expression (77 mg/L)^48^.

### One-step Purification and Immobilization of SrtA onto Magnetic Particle

**Figure 2:**
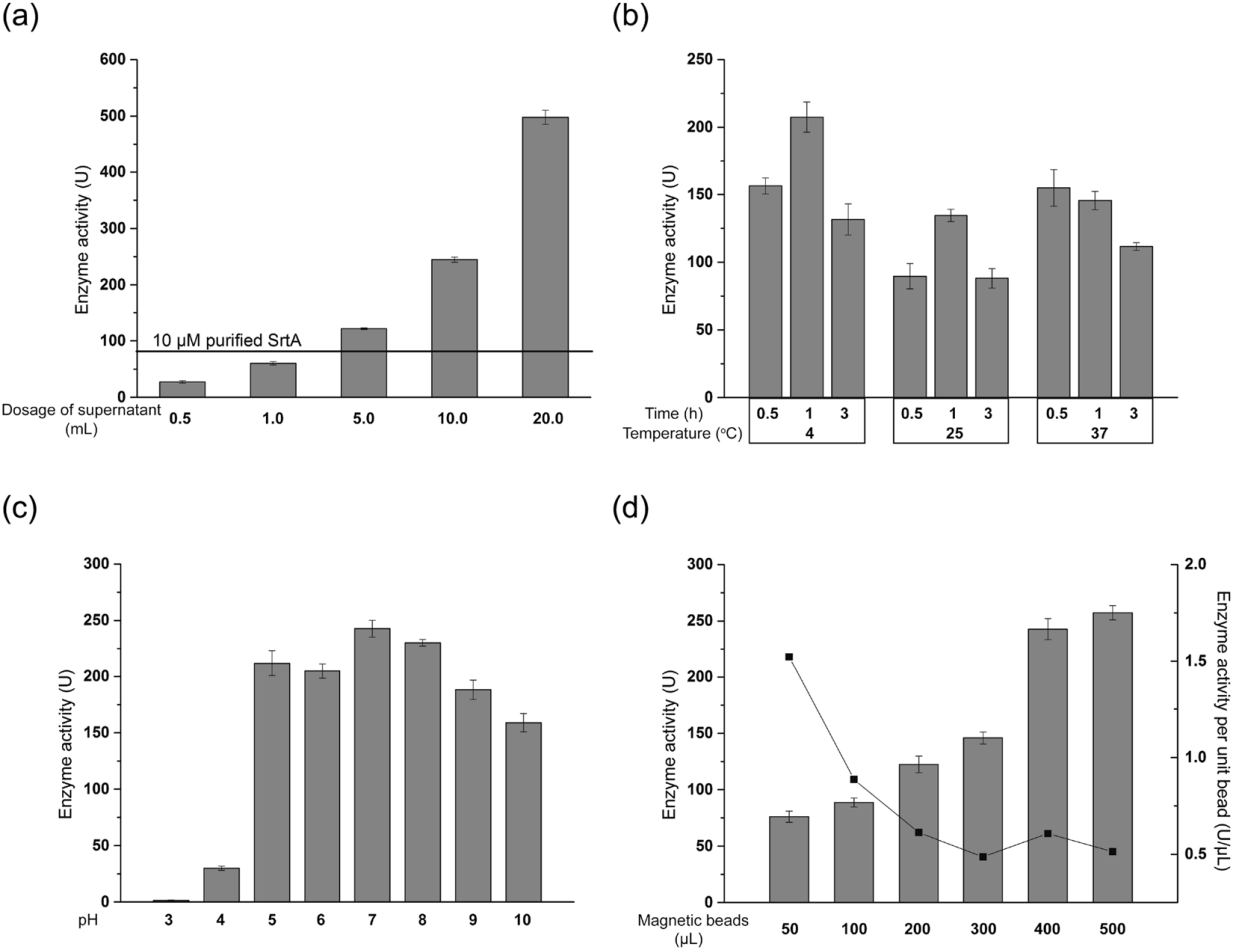
Condition optimization for immobilizing SrtA from the fermentation supernatant to IDA (iminodiacetic acid)-nickel-modified magnetic particles. (**a**) SrtA activity after treatment of different culture supernatant volumes with 500 μL magnetic particles; (**b**) effect of incubation time and temperature on enzymatic activity when incubating 5 mL supernatant with 500 μL magnetic particles; (**c**) effect of culture supernatant pH (from 3 to 10) on enzymatic activity when incubating 5 mL supernatant with 500 μL magnetic particles; and (**d**) specific enzymatic activity after treatment with different magnetic particle volumes with 5 mL culture supernatant at 4 °C for 1 h. All of the experiments were performed in triplicate, and the data are the mean values with the standard deviation.

With secreted SrtA in hand, a one-step protocol for the purification and immobilization of SrtA was investigated. In this case, commercially available IDA-nickel modified magnetic particles were used to immobilize SrtA having an N-terminus His-tag. In SML reactions, generally 10 μM of free SrtA was applied depending on the nature of the protein donor and acceptor. Therefore, the enzymatic activity of free SrtA at 10 μM was measured and used as a reference parameter to optimize SrtA immobilization. First, 500 μL of magnetic particle beads was added to different volumes of fermentation supernatant (0.5, 1.0, 5.0, 10, or 20 mL) and incubated for 1 h at room temperature. MPI-SrtA was separated using a magnetic field and then washed three times with deionized water, followed by enzymatic activity measurements. As shown in Fig. 2a, the enzymatic activity of MPI-SrtA gradually increased as more culture supernatant was used. When 5 mL of supernatant was treated with 500 μL of magnetic beads, the resulting enzymatic activity was 121.7 U, which is higher than the enzymatic activity of 10 μM free SrtA (82.9 U).

Incubation temperature and time might also affect the enzymatic activity and loading efficiency, thus their effects were investigated. Incubating 5 mL of the supernatant with 500 μL of magnetic particles was performed at 4, 25 and 37 °C for 0.5, 1 and 3 h, respectively. Generally, the incubation of magnetic particles with the supernatant for 1 h provided the highest enzymatic activity, whereas further extension of the incubation time to 3 h resulted in decreased activity, likely because of the nonspecific absorption of undesirable proteins onto the particles. As for the incubation temperature, higher enzymatic activity was achieved at 4 °C than that at 25 or 37 °C. Thus, the maximum enzymatic activity (208.1 U) was obtained when immobilization was performed at 4 °C for 1 h (Fig. 2b).

His-tag binding to the nickel resin was strongly influenced by pH values. Therefore, supernatant binding of SrtA to IDA-Nickel modified magnetic particles at pH values ranging from 3 to 10 was also investigated. Under acidic conditions below 4, MPI-SrtA exhibited no or very low enzymatic activity, indicating that SrtA-His binding to Ni^2+^ on magnetic particles was inhibited, probably because the histidine residues of the His-tag were protonated. Increasing the pH of the supernatant from 5 to 7 enhanced MPI-SrtA enzymatic activity, which reached the peak activity of 242.6 U under neutral conditions. Further increase in the supernatant pH from 8 to 10 decreased the enzymatic activity, suggesting decreased amounts of SrtA immobilized onto the magnetic particles. Thus, a pH of 7 was chosen as the optimal condition for SrtA immobilization (Fig. 2c).

The amount of magnetic particles used for immobilization was also optimized at 4 °C under neutral conditions, as this condition will affect the reactant diffusion. After incubating 5 mL of supernatant with 50 to 500 μL of magnetic particles for 1 h, the enzymatic activity was enhanced accordingly, and the highest specific enzyme activity (0.61 U/μL) was obtained when 400 μL of magnetic particles was used. Thus, the optimal condition for constructing MPI-SrtA is as follows: incubation of 400 μL of magnetic particles with 5 mL of fermentation supernatant at 4 °C for 1 h, which generates 243.5 U of specific enzyme activity at 0.61 U/μL (Fig. 2d). To test the scaling ability of this procedure, 20 mL of MPI-SrtA was prepared by incubating 250 mL of supernatant with 20 mL of magnetic particles. The enzymatic activity of MPI-SrtA were 318.2 U at 0.8 U/μL, an approximately 30% improvement compared with the small-scale experiment. The stability experiment demonstrated that MPI-SrtA could be stored at 4 °C for one month without significant activity loss (SI, Fig. S1). After MPI-SrtA was constructed, it was microscopically analyzed. Commercially available IDA-nickel magnetic particles are uniform, with an average diameter of 19 μm. After performing enzyme immobilization, the average diameter of MPI-SrtA increased to 34 μm, indicating successful SrtA immobilization onto the magnetic particles (SI, Fig. S2). In addition, SDS-PAGE was used to further confirm successful immobilization. In this case, MPI-SrtA was washed first with 10 mM of imidazole, which resulted in the release of only trace amounts of SrtA (SI, Fig. S3, line 3). Further washing with 500 mM of imidazole yielded two protein bands that correspond to SrtA, whose expected band is at 17 kDa, and its dimer (SI, Fig. S3, line 4). The formation of SrtA dimer is resulted from a disulfide bridge of two active site Cys184 residues located in enzyme under denaturing conditions without adding reducing reagent^55^. The eluate was dialyzed and lyophilized to yield 407.2 μg of protein, which suggested that 91% of the SrtA was immobilized onto the IDA-nickel magnetic particles according to the amount of SrtA in 5 mL of supernatant (449.0 μg).

### Application of Immobilized SrtA in Peptide to Peptide Ligation

**Figure 3:**
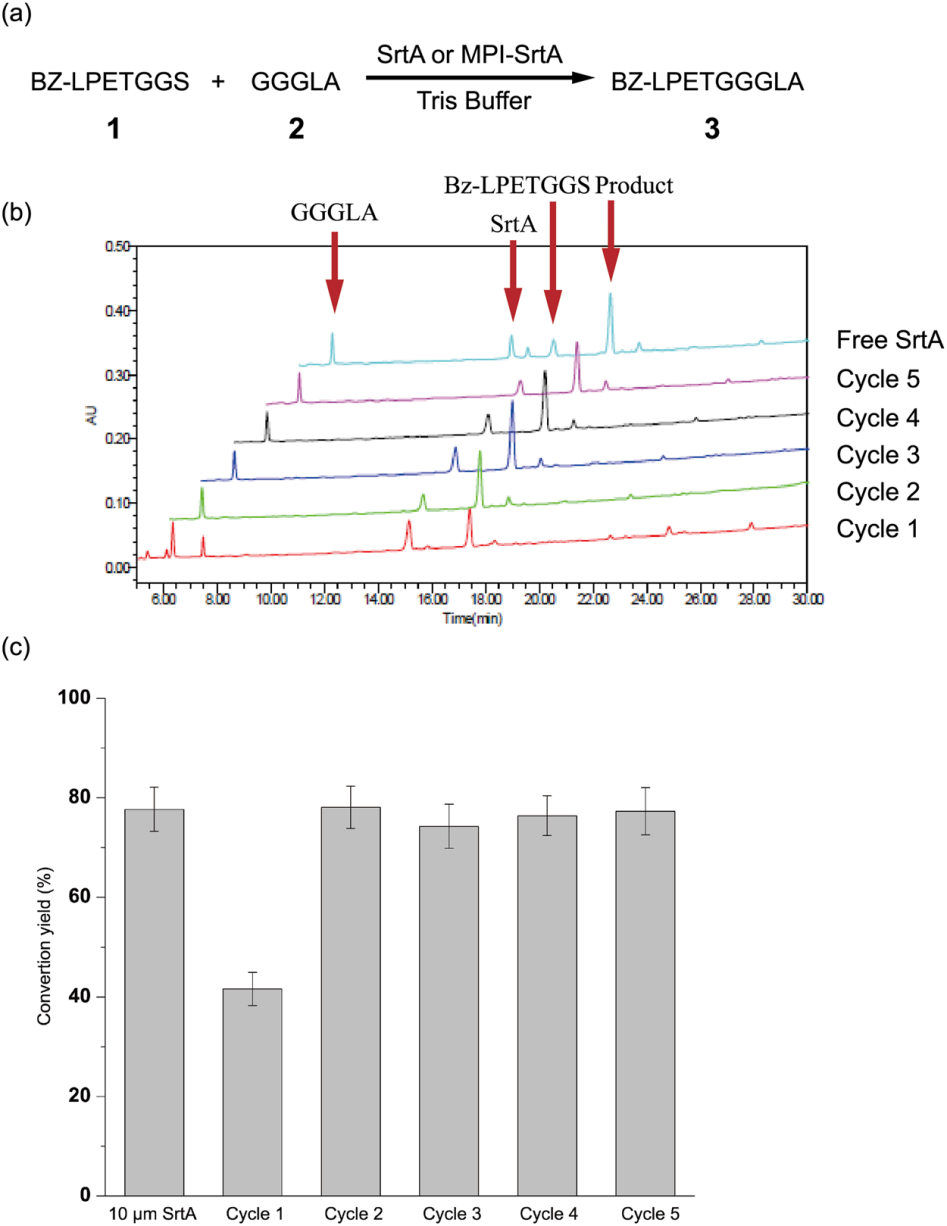
MPI-SrtA- or free SrtA-catalyzed peptide-to-peptide ligation. (**a**) Ligation reaction of peptide Bz-LPETGGS **1** and GGGGLA **2**, conditions: 0.3 M Tris-HCl buffer (pH = 7.5) containing 0.15 M NaCl, 5 mM CaCl_2_, 2 mM 2-mercaptoethanol, 0.125 mM peptide **1** and 0.625 mM peptide **2** in the presence of 100 μL MPI-SrtA or 10 μM free SrtA. (**b**) HPLC diagraph of MPI-SrtA- or free SrtA-catalyzed peptide-to-peptide ligation. (**c**) Reuse and recycling of MPI-SrtA in peptide-to-peptide ligation.

With MPI-SrtA in hand, we examined its efficiency and recyclability to catalyze peptide-peptide ligation, using short peptide 1 and 2 as the donor and acceptor peptides (Fig. 3a), and the reactions were monitored by HPLC and MALDI-TOF MS. For control and comparison purposes, the same reaction was also performed using 10 μM of free SrtA as a catalyst. After incubation at 37 °C for 2.5 h, reaction aliquots were subjected to analytic HPLC using an RP C18 column and MALDI-TOF MS. Both MPI-SrtA and free SrtA catalyzed the peptide-to-peptide ligation successfully. As shown in Fig. 3b, a new peak was produced at a retention time of 17.5 min, which was confirmed to be the expected ligation product 3 by MALDI-TOF MS [M + Na^+^, Calcd. 975.1, Found. 997.4]. The ligation yields were 42% and 78% for MPI-SrtA and free SrtA, respectively. Then, MPI-SrtA was recycled using a simple magnetic field-assisted separation protocol followed by washing with deionized water (1 mL) two times. It was interesting to observe that the ligation yield increased to 78% when the recovered MPI-SrtA was used as the catalyst in the second cycle, which was comparable to the efficiency of free SrtA. It was speculated that the lower catalytic efficiency of MPI-SrtA observed in the first round of reaction was probably because of the structural and dynamics changes of the SrtA active site caused by the immobilization, which may have interrupted Ca^2+^ ion binding to the β6/β7 loop of SrtA critical for the catalytic activity. After incubating MPI-SrtA in a solution containing Ca^2+^ ion for a certain period, the Ca^2+^ ion binding to SrtA and the enzymatic activity was completely recovered. To confirm this speculation, freshly prepared MPI-SrtA was incubated in buffer containing 0.15 M of NaCl, 5 mM of CaCl_2_, 2 mM of 2-mercaptoethanol for 2 h, then peptide 1 and 2 was added into this solution. After 2.5 h, HPLC analysis indicated that the ligation efficiency increased to 73% (SI, Fig. S4). Following the same procedures, MPI-SrtA was recovered and reused for another three cycles of reaction under the same conditions without obvious loss of activity as shown in Fig. 3b and c. These results proved that MPI-SrtA retained full enzymatic activity after immobilization onto magnetic particles. The ligation efficiency of MPI-SrtA was comparable to that of free SrtA. Moreover, using a simple recovery protocol, MPI-SrtA was readily recycled for at least four more rounds of reactions without activity loss.

### Application of Immobilized SrtA in Protein Modification

**Figure 4:**
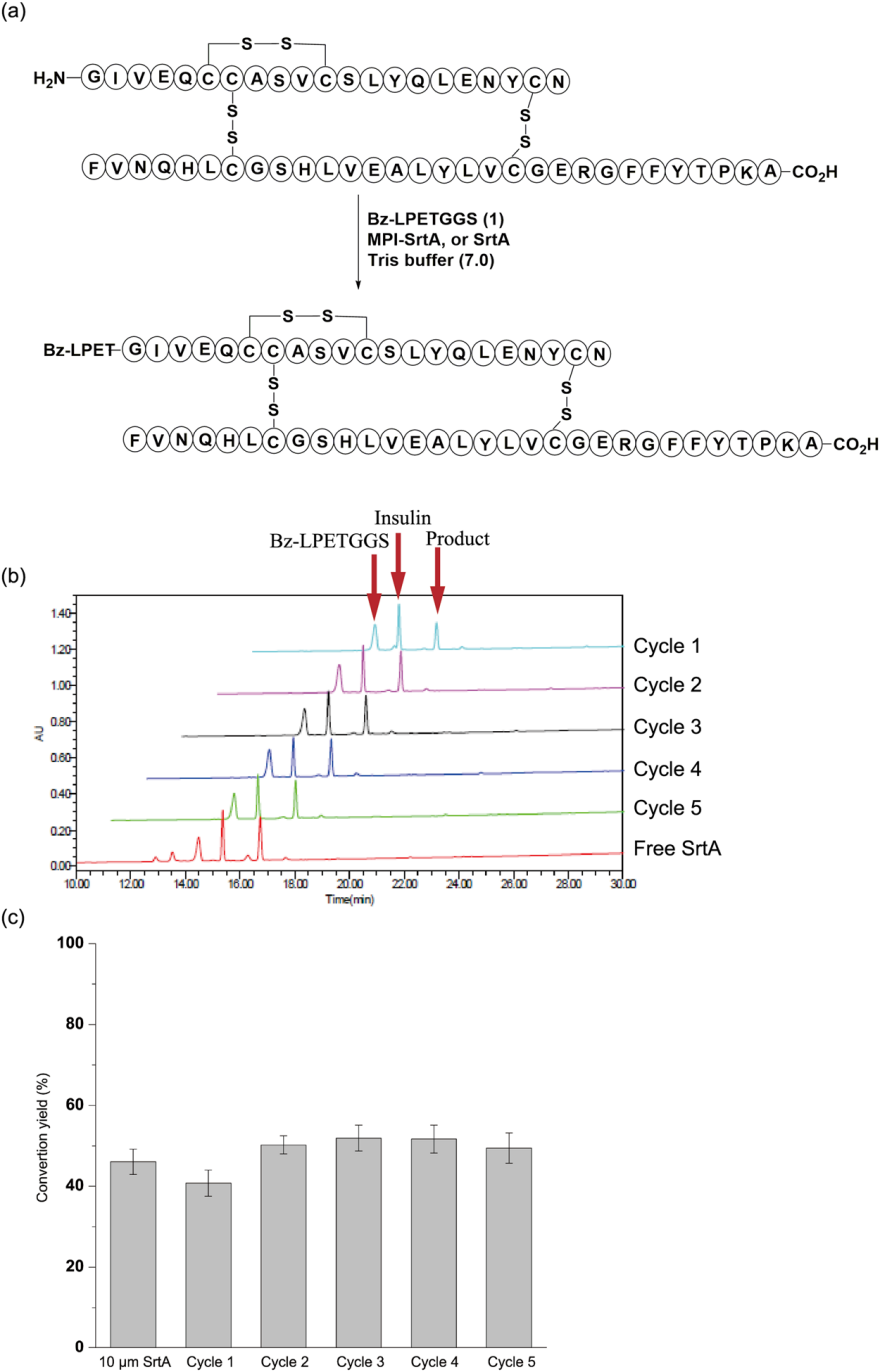
MPI-SrtA- or free SrtA-catalyzed site-specific insulin modification. (**a**) Ligation reaction of peptide Bz-LPETGGS 1 and insulin; conditions: 0.3 M Tris-HCl buffer (pH = 7.5) containing 0.15 M NaCl, 5 mM CaCl_2_, 2 mM 2-mercaptoethanol, 0.125 mM peptide 1 and 2.5 mM insulin in the presence of 100 μL MPI-SrtA or 10 μM free SrtA. (**b**) HPLC diagraph of MPI-SrtA- or free SrtA-catalyzed site-specific insulin modification; (**c**) Reuse and recycling of MPI-SrtA for site-specific insulin modification.

SML is a particularly powerful technology when applied to site-specific protein modification. Therefore, we explored next whether MPI-SrtA could be used to modify proteins. In this case, bovine insulin was used as the model protein, as there is a glycine residue located at its N-terminus that has been established as a nucleophile in SML^56^. Bz-LPETGGS was used as the peptide donor as shown in Fig. 4. After Bz-LPETGGS and insulin were incubated in the presence of MPI-SrtA or free SrtA in Tris-HCl buffer for 12 h, a new peak at a retention time of 16.6 min was formed in both reactions. MALDI-TOF MS detection displayed an m/z of 6325.5, which agrees with the calculated mass of the ligation product Bz-LPETG-insulin (cal. 6300.1). The conversion yield was 41% and 46% for MPI-SrtA and 10 μM of free SrtA, respectively. Following the aforementioned enzyme recovery and recycling protocols, MPI-SrtA was reused for another four cycles, which gave stable conversion yields of approximately 50%. These results demonstrated that MPI-SrtA is also suitable for site-specific protein modification, and it retained almost full reactivity during this biotransformation. The excellent recyclability suggested that the MPI-SrtA separation and recovery processes were highly efficient as well.

## Conclusion

In summary, efficiently secretory expression of SrtA was achieved by using the PelB secretory signal peptide combined with codon optimization and co-expression of molecular chaperones in *E. coli*, which produced 89.9 mg/L of purified SrtA. To the best of our knowledge, this is the first report concerning the extracellular expression of SrtA in *E. coli*, and the expression yield was higher than the highest reported cytoplasmic expression level. Moreover, a simple and efficient strategy was developed and optimized for one-step purification and immobilization of SrtA from the fermentation supernatant based on its His-tag-mediated affinity attachment to IDA-nickel modified magnetic particles. The constructed MPI-SrtA was unambiguously proved to be a robust and recyclable biocatalyst comparable to the free enzyme for peptide-to-peptide ligation and site-specific protein modification. This strategy provides a cost-effective and green approach to generate immobilized biocatalysts for SML, which has great potential for industrial applications.

## Electronic supplementary material

Supporting information
