# Supplementary material for: One-step purification and immobilization of extracellularly expressed sortase A by magnetic particles to develop a robust and recyclable biocatalyst
Source: Sci Rep. 2017 Jul 26;7:6561. doi: 10.1038/s41598-017-06856-y (PMC5529518; doi:10.1038/s41598-017-06856-y)
Supplement: Supplementary file 1 — Supporting information [file 41598_2017_6856_MOESM1_ESM.docx]

**Supporting Information**

**One-step purification and immobilization of extracellularly expressed sortase A by magnetic particles to develop a robust and recyclable biocatalyst**

Xinrui Zhao^a^, Haofei Hong^a^, Xiaozhong Cheng^a^, Shaozhong Liu^a^, Tao Deng^a^, Zhongwu Guo^b^, Zhimeng Wu^a*^

^a^ Key Laboratory of Carbohydrate Chemistry & Biotechnology, Ministry of Education, School of Biotechnology, Jiangnan University, Wuxi, China

^b^ Department of Chemistry, University of Florida, 214 Leigh Hall, Gainesville, Florida 32611, United States of America

^*^Corresponding author:

Zhimeng Wu

Mailing address: School of Biotechnology, Jiangnan University, 1800 Lihu Road, Wuxi, Jiangsu 214122, China.

Tel.: +86-510- 85197582, Fax: +86-510-85197582

E-mail: zwu@jiangnan.edu.

**Table of Contents:**

1. **Strains, chemicals and cultural conditions**.............................................................................S3
2. **Extracellular SrtA enzymatic activity assay**...........................................................................S4
3. **Extracellular expression of SrtA in *E. coli***
   1. **Optimization of signal peptide for extracellular expression of SrtA in *E. coli***....................S4
   2. **Codon optimization and molecular chaperone co-expression for extracellular expression of SrtA in *E. coli***................................................................................................................................S6
4. **General procedure for one-step purification and immobilization of SrtA onto magnetic particles and enzymatic activity arrays**..........................................................................................S8
5. **Peptide 1 and 2 synthesis** .......................................................................................................S10
6. **General procedure for free SrtA or immobilized SrtA catalyzed ligation between BZ-LPETGGS 1 and GGGLA 2** ..................................................................................................S11
7. **General procedure for free SrtA or immobilized SrtA catalyzed ligation between BZ-LPETGGS and insulin** ...........................................................................................................S11
8. **Supporting figures**

**Figure S1:** The stability of immobilized SrtA beads was determined by enzymatic activity assays every 6days ......................................................................................................................................S12

**Figure S2:** The diameter change between original beads and immobilized SrtA bead...................S13

**Figure S3:** The determination of immobilized SrtA by SDS-page analysis ...................................S13

**Figure S4:** HPLC diagraph of ligation of peptide 1 and 2 catalyzed by freshly prepared MPI-SrtA after incubation in buffer .................................................................................................................S14

**Figure S5:** Maldi-Tof Ms of GGGLA ............................................................................................S14

**Figure S6:** Maldi-Tof Ms of Bz-LPETGGS ...................................................................................S15

**Figure S7:** Maldi-Tof Ms of Bz-LPETGGGLA .............................................................................S16

**Figure S8:** Maldi-Tof Ms of Bz-LPETG-Insulin .........................................................................S17

**1. Strains, chemicals and cultural conditions**

The host strain *E. coli* BL21 (DE3) and the vector pET22b were purchased from Novagen (Madison, WI). The commercial plasmids expressing different molecular chaperones (pG-TF2, pTf16, pGro7, pG-KJE8 and pKJE7) were bought from Takara (Dalian, China). The specific substrate of SrtA (Dabcyl-QALPETGEE-Edans) and triglycine peptide were obtained from GL biochem Ltd. (Shanghai, China). The transformed clones were propagated in LB medium (Tryptone 10 g/L, Yeast extract 5 g/L, NaCl 10 g/L) at 37°C for 12 h with constant shaking at 200 rpm. The seed culture (2%) was inoculated into 25 mL TB* medium (Tryptone 12 g/L, glycerol 4g/L, K_2_HPO_4_·3H_2_O 16.4 g/L, KH_2_PO_4_ 2.31 g/L) at 37°C until the OD_600_ reached 0.6 and then incubated with 1 mM IPTG (isopropyl β-D-thiogalactoside) at 25°C for 36 h with constant shaking at 200 rpm. After fermentation broth was centrifuged, the supernatant was collected to measure its enzymatic activity directly and to determine the concentration of SrtA after purification.

Chemical reagents and solvents were purchased from Titan Scientific Lab (Shanghai, China) and used without further purification. Fmoc-protected amino acids and peptide synthesis reagents were purchased from Changzhou Kanglong Biotech Ltd. (Changzhou, China). Analytical RP-HPLC was performed on Waters E2695 with a C18 column. Solid-phase peptide synthesis was performed on a CEM Liberty Blue peptide synthesizer. Peptides were characterized by Maldi-Tof Ms (UltrafleXtreme, Bruker Daltonics; Bremen, Germany).

**2. Extracellular SrtA enzymatic activity assay**

To measure extracellular SrtA activity, specific substrate (Dabcyl-QALPETGEE-Edans) was used. The SrtA enzymatic activity arrays were performed in 200 μL volume of 50 mM Tris-HCl buffer (including 150 mM NaCl, 5 mM CaCl_2_, 5 μg Dabcyl-QALPETGEE-Edans, pH 7.8) and 10 μL extracellular SrtA supernatant. The reactions were carried on at 37^o^C for 1 h with constant shaking at 200 rpm by use of a Synergy H4 hybrid microplate reader (BioTek, Vermont, America) and the flurescence intensity (FI) was detected with 350 nm for excitation and 495 nm for recordings. One unit of SrtA activity was defined as the amount of enzyme (mg) that was able to increase of one FI per minute in the 200 μL reaction mixture. All experiments were performed in triplicate and the mean values were used for further calculations.

**3. Extracellular expression of SrtA in *E. coli***

**3.1 Optimization of signal peptide for extracellular expression of SrtA in *E. coli***

Primers used in vector constructions are listed in **Table 1**. The genome of *S. aureus* (ATCC 35556) was extracted by Genomic Extraction Kit (Qiagen, Valencia, CA) and applied as the template for amplifying Δ59-*srtA*. The gel-purified PCR-amplified Δ59*-srtA* fragments were digested and inserted into the *Nco* I/*Bam* HI site of pET22b. As for the replace of PelB signal peptide in pET22b (pET22b-PelB-SrtA), various signal peptide genes (*ompA*, *torA*, *dmsA* and *fdnG*) were amplified from the genome of *E. coli* and linked into the 5’ terminal of Δ59-*srtA* gene by fusion PCR. The entire fusion PCR fragments were digested and inserted into the *Nde* I/*Bam* HI site of pET22b to construct pET22b-OmpA-SrtA, pET22b-TorA-SrtA, pET22b-DmsA-SrtA and pET22b-FdnG-SrtA. All constructed plasmids were confirmed by DNA sequencing and transformed into *E. coli* BL21 (DE3) strain.

**Table 1 Oligonucleotides used for vector constructions**

| **Gene** | **Forward/**  **Reverse** | **Sequence (5’-3’)** |
| --- | --- | --- |
| Primers for pET22b-PelB-SrtA construction | | |
| Δ59-*srtA* | F | CATGCCATGGAAGCTAAACCTCAAATTCCG |
|  | R | CGCGGATCCTTAGTGGTGGTGATGATGATG  TTTGACTTCTGTAGCTACAAAGAT |
| Primers for signal peptide screening | | |
| OmpA | F | GGAATTCCATATGAAAAAGACAGCTATCGCG |
|  | R | aatttgaggtttagcttgcatGGCCTGCGCTACGGTAG |
| OmpA -SrtA | F | ctaccgtagcgcaggccATGCAAGCTAAACCTCAAATT |
|  | R | CGCGGATCCTTAGTGGTGGTGATGATGATG TTTGACTTCTGTAGCTACAAAGATT |
| TorA | F | GGAATTCCATATGAACAATAACGATCTCTTTCAGG |
|  | R | aatttgaggtttagcttgcatCGCTTGCGCCGCAG |
| TorA-SrtA | F | ctgcggcgcaagcgATGCAAGCTAAACCTCAAATT |
|  | R | CGCGGATCCTTAGTGGTGGTGATGATGATG TTTGACTTCTGTAGCTACAAAGATT |
| DmsA | F | GGAATTCCATATGAAAACGAAAATCCCTGATG |
|  | R | aatttgaggtttagcttgcatAGCGTGCGCAATCCGAC |
| DmsA-SrtA | F | gtcggattgcgcacgctATGCAAGCTAAACCTCAAATT |
|  | R | CGCGGATCCTTAGTGGTGGTGATGATGATG TTTGACTTCTGTAGCTACAAAGATT |
| FdnG | F | GGAATTCCATATGGACGTCAGTCGCAGACA |
|  | R | aatttgaggtttagcttgcatAGCCAGTGCTTGCTTCGG |
| FdnG-SrtA | F | ccgaagcaagcactggctATGCAAGCTAAACCTCAAATT |
|  | R | CGCGGATCCTTAGTGGTGGTGATGATGATG TTTGACTTCTGTAGCTACAAAGATT |

**Table 2 Results of signal peptides mediated extracellular expression of SrtA in *E. coli***

| Signal peptides | Concentration of SrtA (mg/L) | SrtA activity (U/mL) |
| --- | --- | --- |
| Control | 0.5 | 0.3 |
| FdnG | 6.7 | 1.5 |
| OmpA | 12.5 | 3.1 |
| TorA | 14.9 | 3.6 |
| PelB | 30.2 | 9.4 |
| DmsA | 20.6 | 6.8 |

**3.2 Codon optimization and molecular chaperone co-expression for extracellular expression of SrtA in *E. coli***

The frequency of codon usage between SrtA and SrtA* was analyzed by the database (<http://www.kazusa.or.jp/codon>). Primers used in codon optimization are listed in **Table 3**. The codon optimization in Δ59*-srtA* (Ile_158_: ATA→ATT; Arg_99_, Arg_159_: AGA→CGT; Leu_110_, Leu_169_: CTA→CTG; Gly_90_, Gly_119_: GGA→GGC) was carried out by critical annealing temperature-PCR with pET22b-PelB-SrtA as the template DNA ([1](#_ENREF_1)). *E. coli* JM109 was transformed with the mutated plasmid (pET22b-PelB-SrtA*) and the transformants were confirmed by DNA sequencing. The correct plasmid was transformed into *E. coli* BL21 (DE3) strain for SrtA expression.

As for the molecular chaperone co-expression, the *E. coli* BL21 (DE3) strain harboring pET22b-PelB-SrtA* was re-transformed with one of commercial plasmids (pG-TF2, pTf16, pGro7, pG-KJE8 and pKJE7). The steps for the inducible expression of molecular chaperone were performed according to the previously described method [1].

**Table 3 Oligonucleotides used for site-mutations**

| **Gene** | **Forward/**  **Reverse** | **Sequence (5’-3’)** |
| --- | --- | --- |
| Gly_90_: GGA→GGT | F | AGTATATCCAGGTCCAGCAA |
|  | R | TTGCTGGACCTGGATATACT |
| Arg_99_: AGA→CGT | F | ATTAAATCGTGGTGTAAGCT |
|  | R | AGCTTACACCACGATTTAAT |
| Leu_110_: CTA→CTG | F | AAATGAATCACTGGATGATC |
|  | R | GATCATCCAGTGATTCATTT |
| Gly_119_: GGA→GGT | F | AATTGCAGGTCACACTTTCA |
|  | R | TGAAAGTGTGACCTGCAATT |
| Ile_158_: ATA→ATT | F | ACAAGTATTAGAAATGTTAA |
|  | R | TTAACATTTCTAATACTTGT |
| Arg_159_: AGA→CGT | F | AAGTATTCGTAATGTTAAGC |
|  | R | GCTTAACATTACGAATACTT |
| Leu_169_: CTA→CTG | F | AGAAGTTCTGGATGAACAAA |
|  | R | TTTGTTCATCCAGAACTTCT |

**Table 4: Results of codon optimization and co-expression of molecular chaperones mediated extracellular expression of SrtA in *E. coli***

| Strategies | Plasmids | Sortase A | |
| --- | --- | --- | --- |
|  |  | Concentration  (mg/L) | Enzymatic activity  (U/mL) |
| 1. Molecular chaperone  (GroES/GroEL) | pET22b-*srt A**  pGro7 | 55.4 | 12.1 |
| 2. Molecular chaperone  (Tig) | pET22b-*srt A**  pTf16 | 74.2 | 26.5 |
| 3. Molecular chaperone  (GroES/GroEL-Dnak/DnaJ/GrpE) | pET22b-*srt A**  pG-KJE8 | 31.6 | 8.5 |
| 4. Molecular chaperone  (Dnak/DnaJ/GrpE) | pET22b-*srt A**  pKJE7 | 46.8 | 14.4 |
| 5. Molecular chaperone  (GroES/GroEL/Tig) | pET22b-*srt A**  pG-Tf2 | 89.8 | 34.0 |
| 6. Control (without codon optimization and molecular chaperones)  7. codon optimization | pET22b-*srt A*  pET22b-*srt A** | 30.2  42.0 | 9.4  11.7 |

**4: General procedure for one-step purification and immobilization of SrtA onto magnetic particles and enzymatic activity arrays**

The nickel-magnetic beads (400 μL) (Beaver Co., Ltd., Suzhou, China) were pre-washed with 50 mM Tris-HCl buffer (1 mL) three times and shaked with extracellular SrtA supernatant (5 mL) at 4°C for 1 h using a vortex mixer (BE-1100, Qilin Beier Instrument manufacturing Co., Ltd., Haimen, China). Magnetic beads were collected and washed with 2 mL deionized water for three times. Then the immobilized SrtA enzymatic activity arrays were performed in 200 μL volume of 50 mM Tris-HCl buffer (including 150 mM NaCl, 5 mM CaCl_2_, 5 μg Dabcyl-QALPETGEE-Edans, pH 7.8) and a certain amount of immobilized SrtA magnetic beads (50-500 μL). The reactions were carried on at 37^o^C for 1 h with constant shaking at 200 rpm by use of a Synergy H4 hybrid microplate reader (BioTek, Vermont, America) and the fluorescence intensity (FI) was detected with 350 nm for excitation and 495 nm for recordings. All experiments were performed in triplicate and the mean values were used for further calculations.

To prepare 20 mL scale of immobilized SrtA: 20 mL of nickel-magnetic beads were incubated with 250 mL of fermentation supernatant at 4°C for 1 h using a vortex mixer. Enzymatic activity was measured following the above procedure to give 318.2 U with a specific enzyme activity 0.8 U/ uL.

**Table 5 The effect of dosage of supernatant on the activity of immobilized SrtA**

| The dosage of supernatant （mL） | The dosage of magnetic beads  （μL） | Enzymatic activity  (U) |
| --- | --- | --- |
| 10 μM purified SrtA | 0 | 82.9 |
| 0.5 | 500 | 27.4 |
| 1.0 | 500 | 60.3 |
| 5.0 | 500 | 121.7 |
| 10.0 | 500 | 244.8 |
| 20.0 | 500 | 497.9 |

**Table 6 The effect of temperature and time on the activity of immobilized SrtA**

| The dosage of supernatant (mL) | The dosage of magnetic beads (μL) | | Temperature (°C) | Time  (h) | Enzymatic activity (U) |
| --- | --- | --- | --- | --- | --- |
| 5.0 | 500 | 4 | | 0.5 | 156.2 |
| 5.0 | 500 | 4 | | 1 | 208.1 |
| 5.0 | 500 | 4 | | 3 | 131.3 |
| 5.0 | 500 | 4 | | 5 | 164.9 |
| 5.0 | 500 | 25 | | 0.5 | 89.4 |
| 5.0 | 500 | 25 | | 1 | 138.6 |
| 5.0 | 500 | 25 | | 3 | 88.6 |
| 5.0 | 500 | 25 | | 5 | 123.1 |
| 5.0 | 500 | 37 | | 0.5 | 158.7 |
| 5.0 | 500 | 37 | | 1 | 147.2 |
| 5.0 | 500 | 37 | | 3 | 114.8 |
| 5.0 | 500 | 37 | | 5 | 97.3 |

**Table 7 The effect of pH on the activity of immobilized SrtA**

| The dosage of supernatant （mL） | The dosage of magnetic beads (μL) | pH | Enzymatic activity  (U) |
| --- | --- | --- | --- |
| 5.0 | 500 | 3 | 1.4 |
| 5.0 | 500 | 4 | 30 |
| 5.0 | 500 | 5 | 212 |
| 5.0 | 500 | 6 | 205.4 |
| 5.0 | 500 | 7 | 242.6 |
| 5.0 | 500 | 8 | 230.2 |
| 5.0 | 500 | 9 | 188.2 |
| 5.0 | 500 | 10 | 158.2 |

**Table 8 The effect of dosage of magnetic beads on the activity of immobilized SrtA**

| The dosage of supernatant  (mL) | The dosage of magnetic beads  (μL) | Enzymatic activity  (U) |
| --- | --- | --- |
| 5.0 | 50 | 76.1 |
| 5.0 | 100 | 88.6 |
| 5.0 | 200 | 122.6 |
| 5.0 | 300 | 145.7 |
| 5.0 | 400 | 243.5 |
| 5.0 | 500 | 259.6 |

**5. Peptide 1 and 2 synthesis**

The peptides were synthesized on an automatic CEM Liberty Blue peptide synthesizer by Fmoc-chemistry using Fmoc-protected amino acid derivatives. A Rink Amide resin (loading 0.317 mmol/g) was used as the solid support. To synthesize peptides 1 or 2: 5.0 equiv. of amino acids were used in each cycle with the microwave assisted irradiation at 90^o^C, and TBTU (0.5 M in DMF), DIPEA (1.0 M in DMF) (1:1, v/v) were used as the coupling reagent; deprotection of the Fmoc group was carried out using 20% of piperidine in DMF. After the peptides elongation was completed, the peptides were cleaved from the resin by treatment with TFA/*i*-Pr_3_SiH/H_2_O (95:2.5:2.5, v/v/v) followed by precipitation with cold ether. The crude peptide was dissolved in water and lyophilized. The residue was subject to HPLC and Maldi-Tof MS analysis to give Bz-LPETGGS (**1**) and GGGLA (**2**). Both peptides were analyzed by HPLC analysis, the results showed that the final products were >95% pure, which were good enough to use directly.

**6. General procedure for free SrtA or immobilized SrtA catalyzed ligation between BZ-LPETGGS 1 and GGGLA 2**

The reactions between BZ-LPETGGS **1** and GGGLA **2** were performed in 200 μL volume of 300 mM Tris-HCl buffer (including 150 mM NaCl, 10 mM CaCl_2_, pH 7.5), 0.125 mM BZ-LPETGGS, 0.625 mM GGGLA and 10 μM purified SrtA or 100 μL SrtA-immobilized beads. The reactions were carried on at 37^o^C for 2.5 h under vigorous shaking (200 rpm). After reaction was completed, the reaction mixture was analyzed by RP-HPLC using C18 column (5 μm, 4.6 mm ×250 mm) at 40^o^C. HPLC conditions: 10% ACN in H_2_O (both containing 0.1% TFA) to 70% ACN in H_2_O in 30 min; flow rate: 1 mL/min; monitored with UV at 220 nm.

For recycling and reuse of the immobilized SrtA: MPI-SrtA separated by simple magnetic field followed by washing with 2 mL of deionized water. Then it was used for the new batch reactions.

**7. General procedure for free SrtA or immobilized SrtA catalyzed ligation between BZ-LPETGGS and insulin**

The reactions between BZ-LPETGGS and insulin were performed in 200 μL volume of 300 mM Tris-HCl buffer (including 150 mM NaCl, 10 mM CaCl_2_, pH 7.8), 0.5 mM BZ-LPETGGS, 2.5 mM insulin and 10 μM purified SrtA or 100 μL SrtA-immobilized beads. The reactions were carried on at 37^o^C for 4 h under vigorous shaking (200 rpm). After reaction was completed, the reaction mixture was analyzed by RP-HPLC using C18 column (5 μm, 4.6 mm ×250 mm) at 40^o^C. HPLC conditions: 10% ACN in H_2_O (both containing 0.1% TFA) to 70% ACN in H_2_O in 50 min; flow rate: 1 mL/min; monitored with UV at 220 nm.

For recycling and reuse of the immobilized SrtA: MPI-SrtA separated by simple magnetic field followed by washing with 2 mL of deionized water. Then it was used for the new batch reactions.

**8. Supporting figures**


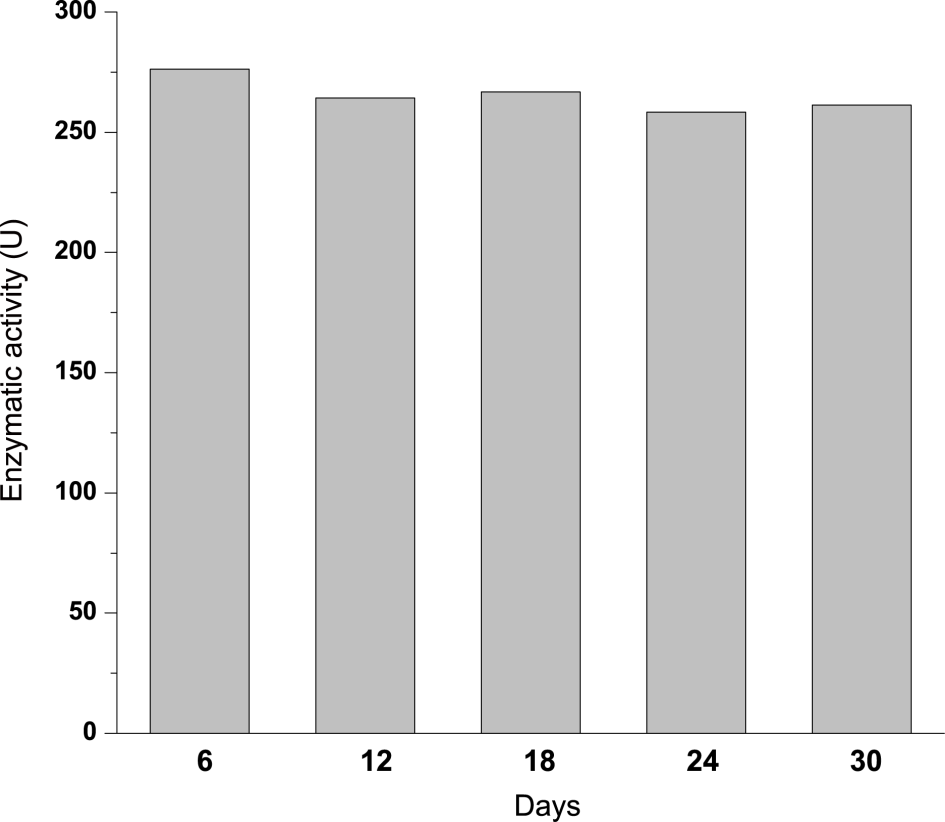


**Figure S1:** The stability of immobilized SrtA beads was determined by enzymatic activity assays every 6 days.


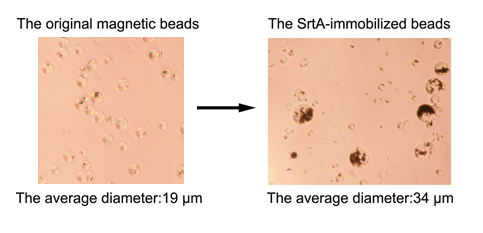


**Figure S2:** The diameter change between original beads and immobilized SrtA beads


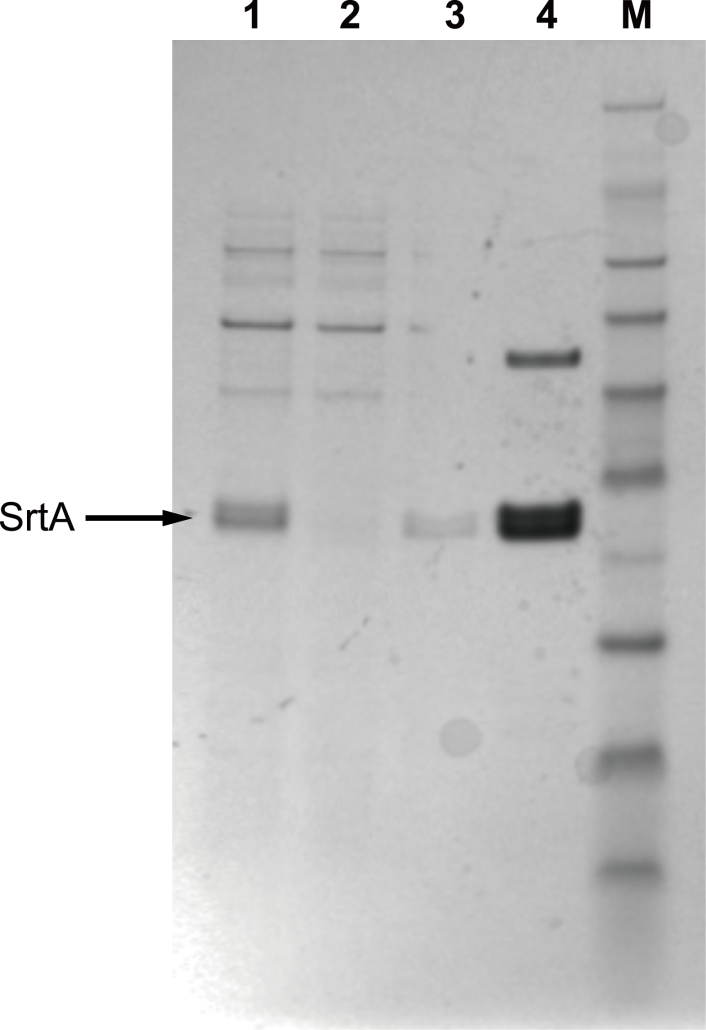


**Figure S3:** The determination of immobilized SrtA by denaturing gel (Thermo Fisher Scientific Inc., NP0342BOX) analysis. Line 1: fermentation supernatant after the expression of SrtA in *E. coli*; Line 2: fermentation supernatant after immobilization with Magnetic beads; Line 3: Magnetic beads elute by washing with 10 mM imidazole (31.4 μg); Line 4: Magnetic beads elute by washing with 500 mM imidazole (407.2 μg).


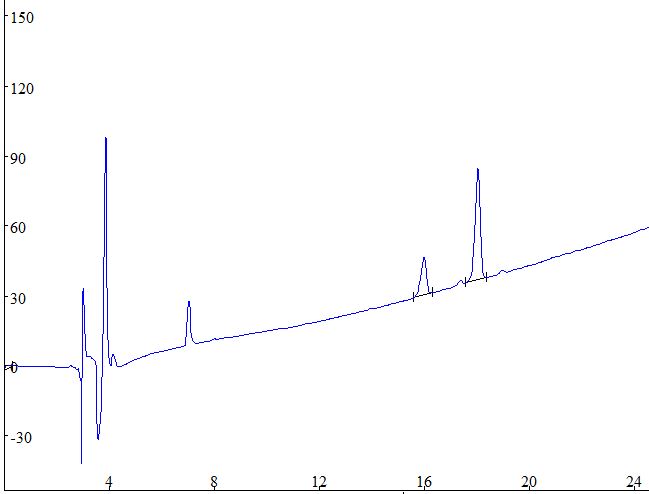


**GGGLA**

**Bz-LPETGGS, 27%**

**Bz-LPETGGGLA, 73%**

**Figure S4:** HPLC diagraph of ligation of peptide 1 and 2 catalyzed by freshly prepared MPI-SrtA after incubation in buffer containing 0.15 M of NaCl, 5 mM of CaCl_2_, 2 mM of 2-mercaptoethanol for 2 h

**Figure S5:** Maldi-Tof Ms of GGGLA, Cal. 430.5, Found, 453.1(M+Na)

**Figure S6:** Maldi-Tof Ms of Bz-LPETGGS, Cal. 820.9, Found, 843.3(M+Na)

**Figure S7:** Maldi-Tof Ms of Bz-LPETGGLA, Cal. 975.1, Found, 997.4(M+Na)

**Figure S8:** Maldi-Tof Ms of Bz-LPET-Insulin, Cal. 6300.7, Found, 6325.6(M+Na)

[1]. Voulgaridou GP, Mantso T, Chlichlia K, Panayiotidis MI, Pappa A. Efficient *E. coli* expression strategies for production of soluble human crystallin ALDH3A1. *PloS one* 2013, **8:**e56582.
